# Extrastriate projections in human optic radiation revealed by fMRI-informed tractography

**DOI:** 10.1007/s00429-014-0799-4

**Published:** 2014-06-06

**Authors:** Ivan Alvarez, D. Samuel Schwarzkopf, Chris A. Clark

**Affiliations:** 1 Institute of Child Health, University College London, London, WC1N 1EH UK; 2 Cognitive, Perceptual and Brain Sciences, University College London, London, WC1H 0AP UK; 3 Imaging and Biophysics Unit, UCL Institute of Child Health, 30 Guilford Street, London, WC1N 1EH UK

**Keywords:** Optic radiation, Visual pathways, Extrastriate, fMRI, Diffusion tensor imaging, Tractography

## Abstract

The human optic radiation (OR) is the main pathway for conveying visual input to occipital cortex, but it is unclear whether it projects beyond primary visual cortex (V1). In this study, we used functional MRI mapping to delineate early visual areas in 30 healthy volunteers and determined the termination area of the OR as reconstructed with diffusion tractography. Direct thalamo-cortical projections to areas V2 and V3 were found in all hemispheres tested, with a distinct anatomical arrangement of superior–inferior fiber placement for dorsal and ventral projections, respectively, and a medio-lateral nesting arrangement for projections to V1, V2 and V3. Finally, segment-specific microstructure was examined, revealing sub-fascicular information. This is to date the first in vivo demonstration of direct extrastriate projections of the OR in humans.

## Introduction

The optic radiation (OR) is the most prominent white matter relay in the visual system, conveying information from the lateral geniculate nucleus (LGN) to the occipital cortex. There has been considerable interest in the neuroanatomy and organization of the OR, as it remains an area of specific risk in resection of the anterior temporal lobe (Sincoff et al. 2004). Such resections can lead to unintended damage to the OR, leading to visual field defects in 48–100 % of patients undergoing anterior temporal lobe resections (Winston et al. 2011).

The human OR has been classically described in post-mortem dissections (e.g., Ebeling and Reulen 1988) and histological slices (Wahler-Lück et al. 1991; Bürgel et al. 1999), and its anatomical course is well documented. Originating at the LGN, the OR is divided into three distinct fiber bundles; anterior, central and posterior. The anterior bundle runs anteriorly above the temporal horn of the lateral ventricle, forming Meyer’s loop over the temporal lobe before turning posteriorly. The central bundle projects laterally from thalamus across the roof of the temporal horn. The posterior bundle runs directly posterior from LGN and joins the other bundles, with all three blending at the level of the sagittal stratum (Peuskens et al. 2004). The merged fibers then run posteriorly and terminate in the vicinity of the calcarine sulcus (Rubino et al. 2005). These three anatomical bundles convey information of different portions of the visual field, with the anterior, central and posterior bundle representing the upper, central and lower visual field, respectively (Peltier et al. 2006; Pujari et al. 2008; Párraga et al. 2012).

More recently, the OR has also been visualized in vivo using diffusion MRI tractography (Basser et al. 2000). Early reports implementing diffusion tensor methods reconstructed the bundle division of the OR (Catani et al. 2003), while more recent work using probabilistic approaches showed reliable estimation of the course of the OR in agreement with post-mortem descriptions (Sherbondy et al. 2008; Clatworthy et al. 2010; Hofer et al. 2010). Tractography reconstructions of the OR have proven useful beyond providing an anatomical description, particularly in presurgical planning (Powell et al. 2005; Yogarajah et al. 2009) but also in intra-operative image guidance (Daga et al. 2012; Winston et al. 2012), and for studying microstructural changes in disease (Bridge et al. 2009; El-Rafei et al. 2011; Kolbe et al. 2011; Groppo et al. 2012).

Despite the growing interest, little work has been conducted exploring the functional significance of white matter fibers—i.e., what representations do distinct fiber populations carry from one region to another (but see Dougherty et al. 2005; Kim et al. 2006; Saenz et al. 2010). In the case of the OR, spatial visual information is highly segregated; both the LGN (Chen et al. 1999; Schneider et al. 2004) and occipital cortex (Tootell et al. 1998; Wandell et al. 2011) follow a retinotopic organization with direct connections between homologous representations of visual space (e.g., Reid and Alonso 1995). As a consequence, damage to specific portions of the OR leads to blindness in a specific segment of the visual field, such as hemianopsia or quadrantanopsia (e.g., Barton et al. 2005).

While the functional segregation of visual field quadrants in OR bundles is well established, it remains unclear whether these projections reach beyond primary visual cortex (V1). In animal models such as the cat (Garey and Powell 1971; Maciewicz 1975) and flying fox (Manger and Rosa 2005), LGN projections to areas V2 and V3 are well established in tracer studies. In the macaque, anterograde and retrograde tracer evidence suggest a direct projection to extrastriate areas (Yukie and Iwai 1981), specifically areas 18 and 19 (Benevento and Yoshida 1981; Fries 1981). While this hypothesis has been contested by negative findings (Yoshida and Benevento 1981; Benevento and Standage 1982), later experiments using fluorescent dyes support the presence of a direct LGN to V2 projection (Bullier and Kennedy 1983; Kennedy and Bullier 1985). The intriguing possibility of a direct extrastriate projection in the macaque, the predominant model species used to inform our understanding of visual system architecture, begets the question of whether this organization also exists in the human. This study presents the first in vivo evidence for direct OR projections to regions V2 and V3 in human occipital cortex using diffusion MRI tractography.

## Methods

### Participants

Thirty healthy adults (12 males, age range 21–35) took part in the study. All participants had normal or corrected-to-normal visual acuity and provided written informed consent. This study was approved by the UCL Research Ethics Committee (London, UK).

### Behavioral measures

In order to assess hand dominance, we used the Edinburgh Handedness Inventory, a 10-item self-report questionnaire probing the lateralization of hand use in everyday tasks (Oldfield 1971). Hand dominance scores were rated on a scale between −100 and 100, with negative numbers indicating a tendency toward left-handedness and positive numbers a tendency toward right-handedness. Participants scored an average of 84.76 ± 6.54 SEM, with 27 participants identified as right-handers (scoring over 50) and 1 participant identified as left-handed (scoring under −50).

Ocular dominance was assessed using the hole-in-card test, also known as the Dolman method (Seijas et al. 2007). The participants held a 300 × 220 mm card at arms length and view a 130 × 130 mm object, in this case a painting, at approximately 3 m distance through the 25 mm hole on the center of the card. The subject alternatively occluded one eye to establish which eye is aligned with the distant object. The eye that upon occlusion causes the image of the target to disappear is considered the dominant eye. While the hole-in-card shows only moderate agreement with alternative tests of ocular dominance (Kommerell et al. 2003), it has high test–retest reliability (Rice et al. 2008) and was preferred due to its widespread use in the literature. Eye dominance as assessed with the hole-in-card test was a nominal measurement, with 14 left eye dominant and 16 right eye dominant participants. No significant correlation between demographic factors (age, gender) and behavioral measures (hand and eye dominance) were observed (Pearson’s *r*, all pairwise comparisons *p* > .05).

### Visual stimulation

Stimuli were generated in MATLAB (v8.0, Mathworks Inc., Natick, MA, USA) using Psychtoolbox (v3.0, Brainard 1997; Pelli 1997) and displayed on an MRI-compatible LCD monitor. The participant viewed the monitor through a mirror while laying supine in the bore of the scanner. The visual display subtended 10.40° of visual angle from fixation.

The stimulus consisted of a 10.40°-radius disk of a dynamic, high-contrast tessellated pseudo-checkerboard with a drifting ‘ripple-like’ pattern that varied across time in spatial frequency and phase. This broadband pattern ensured effective stimulation of visually responsive neurons in visual cortex. Mean luminance of the stimulus was 132.29 cd/m^2^.

The stimulus was presented on an equiluminant gray background through a series of sequential apertures in two conditions. First, a mapping stimulus comprised simultaneous ‘wedge’ and ‘ring’ apertures—a wedge section of 17.14° angle rotated clockwise or counter-clockwise along the polar dimension while a ring section expanded or contracted eccentrically, scaling logarithmically between 0.11° and 4.62° width span. Apertures changed position every 2.376 s, on the onset of each EPI volume acquired. Both apertures cycled at independent frequencies: 21 and 15 volumes per revolution, respectively. A single run consisted of 10 wedge and 14 ring revolutions, followed by 24 volumes of mean luminance, totaling 238 volumes per run. Two runs were conducted, the first with clockwise wedge and expanding ring motion and the second with counter-clockwise wedge and contracting ring motion.

The second condition consisted of a short photic burst of a circular 10.40°-radius aperture of the same pattern, presented for 1 volume and followed by 14 volumes of equiluminant gray background. Ten runs were conducted, totaling 150 volumes acquired.

Throughout both conditions, a 0.30° black center-point cross was presented to aid fixation. Participants were instructed to attend and respond when the cross flashed in red, which occurred in a semi-randomized fashion, with 100–150 events per run, each lasting 200 ms. Participant responses were recorded via an electronic response button and monitored to ensure engagement with the task, and therefore consistent eye fixation.

### MRI acquisition

MR images were acquired on a 1.5 T Avanto MRI system using a 32-channel head coil (Siemens Healthcare, Erlangen, Germany). The experimental session was divided into two consecutive sections taking place on the same day. For the first section only, the bottom elements of the head coil were used, to avoid visual field restrictions. A gradient-echo EPI sequence was used (TR = 2,376 ms, TE = 30 ms, 33 ascending slices, 18 % inter-slice gap), with off-axial acquisitions maximizing occipital lobe coverage and effective resolution of 3 mm^3^ isotropic voxels for 4 subjects and 3.3 mm^3^ isotropic voxels for 26 subjects. A T1-weighted anatomical image (TR = 11 ms, TE = 4.94 ms resolution = 1 mm isotropic) was acquired in-plane with the functional images to aid registration, and B0 field maps (TR = 487 ms, TE_1_ = 5.28 ms, TE_2_ = 10.04 ms) were acquired to estimate local field inhomogeneities. For the second section, the full head coil arrangement was used and a further anatomical T1-weighted volume (TR = 11 ms, TE = 4.94 ms, resolution = 1 mm isotropic) was acquired to reconstruct the cortical surface, as well as a diffusion-weighted EPI protocol with 60 non-collinear gradient directions at *b* = 1,000 s/mm^2^ and 3 *b* = 0 s mm^2^ reference images (TR = 7,300 ms, TE = 81 ms, maximum gradient amplitude = 40 mT/m^−1^, 60 slices, resolution = 2.5 mm isotropic).

### Functional imaging analysis

T1-weighted anatomical images were processed with FreeSurfer (Dale et al. 1999; Fischl et al. 1999). Volumes underwent automated segmentation to generate gray and white matter boundaries, and the gray matter surface reconstructed to create a smooth two-dimensional representation of cortex.

Functional EPI images were pre-processed in SPM8 (Wellcome Trust Centre for Neuroimaging, http://www.fil.ion.ucl.ac.uk/spm). All images were bias-corrected, realigned to the first image of each run to reduce movement artifacts, unwrapped to correct for field inhomogeneities and slice timing-corrected to reduce variation in the time series introduced by the timing of the slice acquisition. Finally, a two-step co-registration was performed on all functional images, by first registering to the in-plane anatomical T1-weighted image and then registered to the anatomical image acquired with the full 32-channel head coil array, in order to maximize registration precision.

fMRI data from the mapping condition were modeled using a population receptive field (pRF) model using in-house software in MATLAB. Briefly, model predictions were based on the a priori knowledge of stimulus position during stimulation and the assumption of single Gaussian receptive field (Dumoulin and Wandell 2008). This model prediction was then convolved with the hemodynamic response functions (HRF), individually estimated for each subject by fitting the time series acquired during photic burst stimulation with a double gamma function (Friston et al. 1995). Mapping data were sampled from volume space to the cortical surface reconstructions, spatially smoothed (FWHM = 8.3 mm) and compared to the model predictions via Pearson’s correlation coefficient. Parameter values of the predictive model that yielded the highest correlation were then used as the starting point for fitting the un-smoothed mapping data, using the Nelder–Mead algorithm for unconstrained nonlinear minimization (Lagarias et al. 1998). The winning model outputs were then smoothed across the cortical surface (FWHM = 5 mm) and projected onto the inflated cortical surface.

Polar angle estimates derived from the pRF model were displayed on the reconstructed cortical surface to delineate region boundaries between primary visual cortex (V1), areas V2 and V3 according to their retinotopic organization (Sereno et al. 1995; DeYoe et al. 1996; Engel et al. 1997). Dorsal and ventral components of these areas were identified, creating six regions per hemisphere sampled: V1d, V1v, V2d, V2v, V3d and V3v. One additional region, V3A on the anterolateral boundary of dorsal V2, was delineated as a control area. Region definitions were then transformed into volume space to inform tractography.

### Diffusion imaging analysis

Diffusion-weighted images were registered to the first, non-diffusion-weighted volume (*b* = 0) to correct for head motion and eddy-current artifacts using FSL-FDT (Behrens et al. 2003). Data were then analyzed with MRtrix (Tournier et al. 2012) by fitting a diffusion tensor model and calculating fractional anisotropy (FA) and mean diffusivity (MD) maps. FA maps were subsequently thresholded to retain values at or above 0.7 and spatially eroded to create a white matter skeleton mask. Voxels retained in the white matter skeleton were used to estimate the diffusion response function. The full diffusion data were then fitted with a constrained spherical deconvolution model (maximum harmonic order = 8) to produce a multi-fiber model of white matter diffusion at each voxel.

In order to reconstruct the path of the OR, a seed-to-target tractography approach was used. The LGN was manually defined on T1-weighted images for each participant, based on anatomical markers (Fujita et al. 2001) and used as ‘seed’ regions. Manual delineations aimed at minimizing contributions from neighboring subcortical structures, including the pulvinar and superior colliculus, therefore a conservative definition of the LGN was preferred. Functionally defined visual maps were registered to diffusion space, and a single ROI consisting of voxels belonging to either area V1, V2 or V3 was used as a single target region in probabilistic tractography (step size = 0.2 mm, angular threshold = 1 mm, FA threshold = 0.1, streamlines = 10,000). This procedure was performed independently for each hemisphere, in each subject. A single target ROI approach was adopted in order to ensure unbiased sampling of the cortical targets, allowing the estimation of the number of streamlines terminating in each target area, rather than defining said number a priori by tracking each target region individually. The direction of tracking, thalamus to occipital, ensured an accurate reconstruction of the highly angular path of the OR at the level of Meyer’s loop.

Resulting OR streamlines were displayed in TrackVis (http://www.trackvis.org) and manually constrained to reject anatomically incorrect streamlines. The following heuristic rules were applied; streamlines were rejected if they were (a) crossing to the contralateral hemisphere, (b) projecting superiorly from the LGN, (c) projecting antero-inferiorly, into the temporal pole and (d) projecting parallel to the medial wall of the posterior horn of the lateral ventricle. An average of 67 % of streamlines were rejected [mean number of streamlines rejected = 6,738 (67 %) ± 1,341 (13 %) SEM]. This heuristic approach constrained the tractography to the known anatomical course of the OR (Ebeling and Reulen 1988; Párraga et al. 2012), with the aim of increasing the confidence on the virtual reconstruction of the white matter tract and ensuring accurate sampling of microstructural parameters. The procedure was carried out independently for each hemisphere analyzed.

### Tract segmentation

OR tractography results were segmented using two different schemes; one constrained by the representations of upper and lower visual field (visual field-based) and one based on the visual map hierarchy (hierarchy-based). In both cases, a Boolean approach was implemented, using the inclusion (AND) and exclusion (NOT) criteria defined below.

For visual field-based segmentations, streamlines in the OR terminating in dorsal visual areas, representing the lower visual field (V1d, V2d, V3d), were classified as belonging to the ‘dorsal’ segment, while streamlines terminating in ventral areas, representing the upper visual field (V1v, V2v, V3v), were classified as part of the ‘ventral’ segment. In both cases, streamlines common to both upper and lower visual field representations were excluded.

For hierarchy-based segmentations, streamlines were divided into those projecting exclusively to each of the following cortical areas: V1, V2 and V3. For example, the V1 projection segment consisted of streamlines projecting to either V1d or V1v, but not V2 or V3 maps. In all cases, dorsal and ventral components were combined.

In summary, two segmentation schemes were applied to the OR in each hemisphere, resulting in five segments: dorsal, ventral, V1, V2 and V3 projections of the OR.

### V3A control condition

An additional condition with a control ROI was carried out in order to estimate the rate of acceptance of invalid streamlines, as an approximation of the false-positive rate in the probabilistic tractography method implemented. Area V3A was chosen as the control ROI, as no direct thalamic projection was expected based on the human or macaque literature. Tractography was performed between the LGN and a single target region comprising areas V1, V2, V3 and V3A. The ratio of streamlines terminating in the control region against those terminating in areas V1, V2 or V3 was calculated and used as an estimate of the false-positive rate of the method.

## Results

### Anatomy of the OR

Both striate and extrastriate projections of the OR were identified in all 60 hemispheres tested. Key structural characteristics were observed for both segmentation schemes, which are described in turn.

#### Visual field-based segmentation

**Fig. 1:**
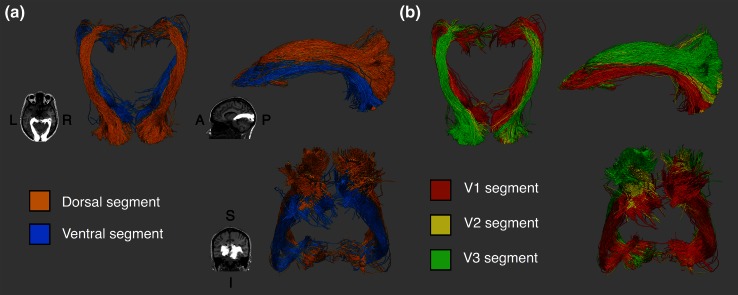
Segments of the optic radiation in a representative participant. **a** Visual field-based segmentation, with dorsal segments following a superior profile, and ventral streamlines populating the more inferior and lateral parts of the tracts. **b** Hierarchy-based segmentation, with *V1*, *V2* and *V3* projections displayed. *V1* streamlines form the most medial segment, while *V2* and *V3* form a nesting pattern dorso–laterally

Dorsal and ventral segments of the OR follow parallel, but distinct anatomical courses (see Fig. 1). The dorsal segment projects laterally from the LGN and maintains a superior profile for the rest of its posterior course toward the occipital lobe. The ventral segment traces a similar anterior path, covering both the anterior and central bundles including Meyer’s loop, but then follows an inferior profile spanning the whole anterior–posterior route. From an axial vantage point, the dorsal segment fans out more laterally as it approaches occipital territories, while the ventral segment retains a medial profile. This anatomical arrangement was observed across subjects, with the superior–inferior division of the long segment of the OR being a marked feature visible in all hemispheres tested (see "Appendix 1" for segment renders of all subjects).

#### Hierarchy-based segmentation

**Fig. 2:**
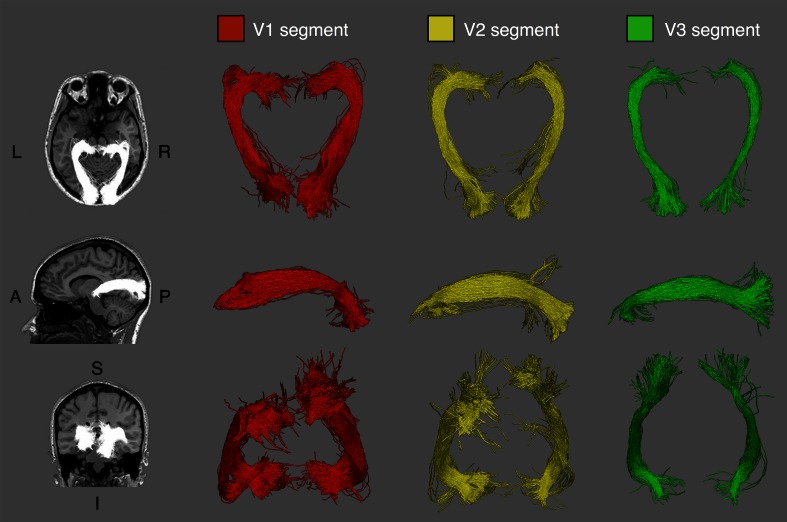
Hierarchy-based segmentation of the optic radiation in a representative participant. Segments terminating in regions *V1*, *V2* and *V3* are displayed, demonstrating the anatomical course of each segment

**Fig. 3:**
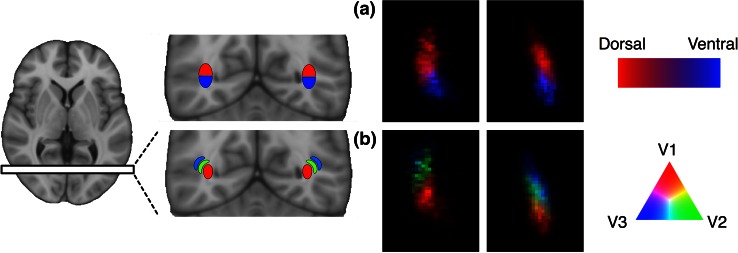
Visitation maps for optic radiation tractography in a representative participant, visualized on a single coronal slice immediately posterior to the lateral ventricle. **a** Visual field-based segmentation shows the superior–inferior division of streamlines, in *red* and *blue*. **b** Hierarchy-based segmentation discriminates streamlines into dorso–lateral nesting compartments, in *red*, *green* and *blue*

The arrangement of V1, V2 and V3 segments follows a media-lateral nesting pattern, with the V1 segment at its center, surrounded by the V2 segment, which in turn is surrounded by V3 (Fig. 2). All three segments follow a similar curved course, with the V1 segment being the most medial part of the tract, and V2 and V3 segments subsequently more lateral (see Fig. 3). It is immediately apparent that the V1 segment is substantially larger than the V2 or V3 segments, which is in agreement with the existing idea of the OR as a primarily striate projection and also the larger cortical area span of V1 compared to V2 or V3 (Yan et al. 2009). The overall nesting arrangement was again observed in all tested hemispheres (for visitation maps of all subjects, see "Appendix 2").

### Variability in OR microstructure

Microstructural markers were sampled by identifying the voxels traversed by all streamlines belonging to each tract segment and computing the mean value for FA and MD in those voxels for each hemisphere. The number of streamlines retained in each tract segment was also extracted as a relative measure of segment size.

In order to test whether microstructural markers varied between visual field-based (dorsal, ventral) and hierarchical-based (V1, V2, V3) segments of the OR, we employed a series of mixed factorial ANOVA tests. Univariate models were preferred due to the exploratory nature of the study. All statistical analyses were carried out in SPSS (v21, IBM SPSS Statistics, IBM Corp., Armonk, New York, USA).

#### Effect of covariates

In order to test the effects of hierarchical segmentation scheme on outcome measures, we performed three independent mixed factorial ANOVAs, one for each outcome measure (FA, MD, streamline count). The ANOVA model incorporated hierarchical segmentation scheme and hemisphere as within-subject variables and age, gender, hand dominance and eye dominance as between-subject variables. These tests revealed no significant main effects or interactions (all effects *p* > .05) of any between-subject variables, namely age, gender, hand dominance or eye dominance. Therefore, in order to increase statistical power, the between-subject variables were dropped and a restricted model, only incorporating the within-subject variables (segmentation scheme and hemisphere) is presented here.

#### Visual field-based segmentation

**Fig. 4:**
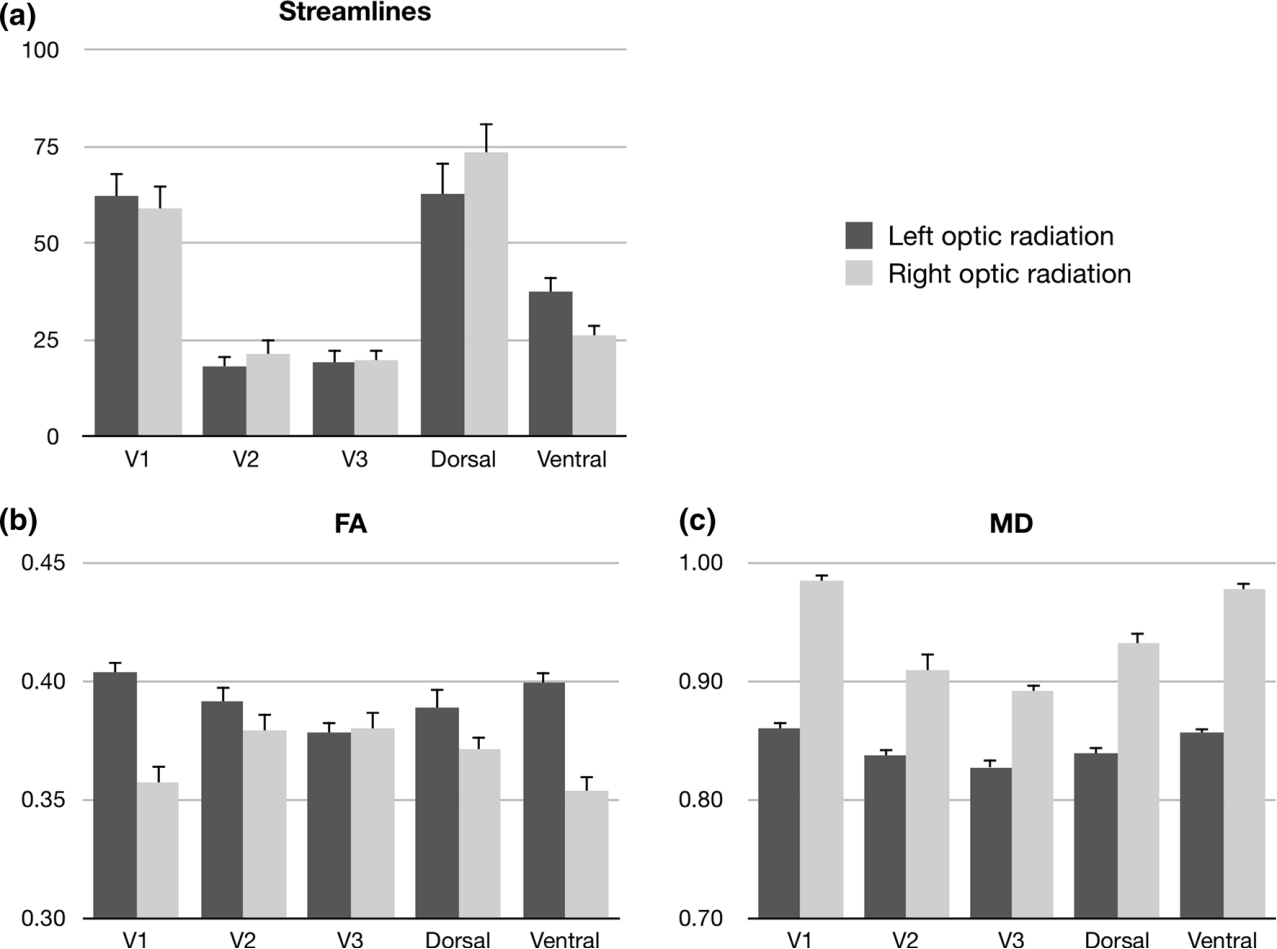
Microstructural values across thirty participants for hierarchy-based segmentation (*V1*, *V2*, *V3*) of the optic radiation. Values displayed are **a** number of streamlines as a percentage of all streamlines in the optic radiation, **b** mean fractional anisotropy (*FA*) and **c** mean diffusivity (*MD*). Percentage streamlines for each hemisphere total 100 %

Three mixed factorial ANOVA models were tested, examining the effect of visual field-based segmentation on the outcome measures described above. A significant effect of segmentation was found on MD (*F* = 29.96, *df* = 1, 29, *p* < .001) but not FA (*F* = 1.80, *df* = 1, 29, *p* = .191), with MD being higher in the ventral compared to the dorsal segment. For both MD and FA, a significant effect of hemisphere was observed (MD: *F* = 102.84, *df* = 1, 29, *p* < .001; FA: *F* = 40.96, *df* = 1, 29, *p* < .001), with a general tendency toward lower microstructural content in the right hemisphere (reduced FA, increased MD; see Fig. 4).

The increase in mean diffusivity in the ventral segment can be partially attributed to the anatomical course of the two segments described above; with the ventral segment being more proximal to the posterior horn of the lateral ventricle compared to the dorsal segment. It is possible that a partial volume effect with adjacent cerebral spinal fluid influenced the increased diffusivity in the ventral segment. Nevertheless, the lack of concomitant differences in FA between segments suggest other factors, as cerebral spinal fluid contamination typically leads to decreased FA, so microstructural differences between these two segments must be interpreted with caution.

In addition to microstructural markers, a significant effect of segmentation on streamline count was found (*F* = 25.04, *df* = 1, 29, *p* < .001), with a larger streamline count in dorsal segments compared to ventral segments (Fig. 4), independent of hemisphere (*F* = 4.09, *df* = 1, 29, *p* = .052).

#### Hierarchy-based segmentation

Three mixed factorial ANOVA models were tested, examining the effect of hierarchy-based segmentation and hemisphere on FA, MD and streamline count. A significant effect of segmentation was found on both MD (*F* = 92.74, *df* = 2, 58, *p* < .001) and FA (*F* = 4.52, *df* = 2, 58, *p* < .05), with significant interactions of segmentation with hemisphere for both microstructural measures (MD: *F* = 34.78, *df* = 2, 58, *p* < .001; FA: *F* = 48.01, *df* = 2, 58, *p* < .001). To further investigate the interaction terms, post hoc paired *t* tests were performed, revealing differential effects of hemisphere on segment microstructure (Fig. 4). Bonferroni correction for multiple comparisons was applied across all subsequent post hoc tests.

For FA, values decreased with cortical hierarchy in the left hemisphere, with significant step effects between V1 and V2 segments (*t* = 5.96, *df* = 29, *p*_corr_ < .001) and between V2 and V3 (*t* = 8.78, *df* = 29, *p*_corr_ < .001). In comparison, the right hemisphere exhibited the opposite effect, with an increase in FA between V1 and V2 segments (*t* = 4.88, *df* = 29, *p*_corr_ < .001) but no significant difference between V2 and V3 segments (*t* = 0.36, *df* = 29, *p*_corr_ = .999).

In the case of MD, a reduction of MD was observed with increasing cortical hierarchy, both in left (V1–V2: *t* = 6.46, *df* = 29, *p*_corr_ < .001; V2–V3: *t* = 3.23, *df* = 29, *p*_corr_ < .05) and right ORs (V1–V2: *t* = 8.64, *df* = 29, *p*_corr_ < .001; V2–V3: *t* = 3.53, *df* = 29, *p*_corr_ < .05).

Finally, the effect of hierarchy-based segmentation on streamline count was assessed, with a significant effect of segmentation (*F* = 42.88, *df* = 2, 58, *p* < .001) and no interaction with hemisphere (*F* = 0.22, *df* = 2, 58, *p* = .800). Within each hemisphere, a larger number of streamlines were found in the V1 segment compared to the V2 (*t* = 8.04, *df* = 59, *p*_corr_ < .001) or V3 segments (*t* = 8.15, *df* = 59, *p*_corr_ < .001). No significant difference between V2 and V3 segments was found (*t* = 0.09, *df* = 59, *p*_corr_ = .930).

**Fig. 5:**
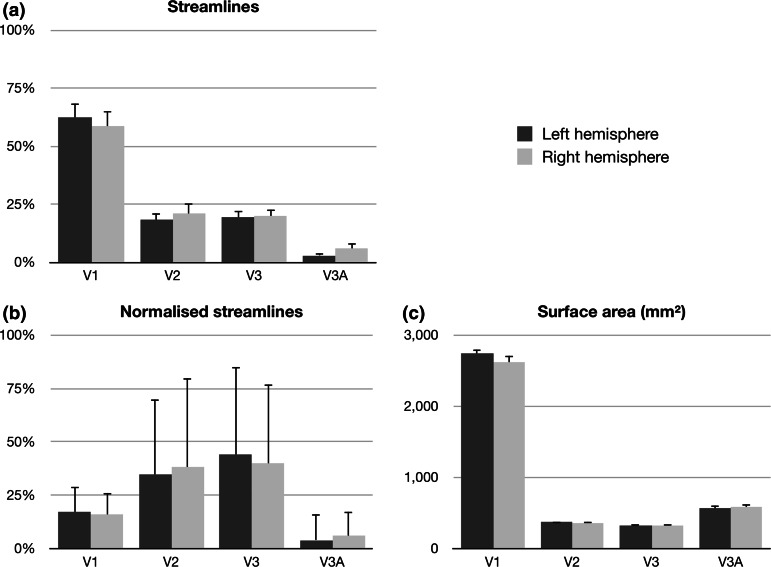
Streamline counts for three hierarchy-based segmentations of the optic radiation (*V1*, *V2*, *V3*) and one control region, *V3A*. Values displayed are **a** number of streamlines as a percentage of all streamlines in the optic radiation, **b** number of streamlines normalized by the surface area of the target region and **c** the surface area of the target region, in mm^2^. Percentage streamlines for each hemisphere total 100 %

In order to assess whether the effect of hierarchy-based segmentation on streamline count was driven by the difference in size between target regions, we normalized the streamline counts by dividing each seed-target pair (V1, V2 and V3) by the surface area (mm^2^) of its corresponding target region, independently for each hemisphere (see Fig. 5). A main effect of segmentation was again observed (*F* = 10.91, *df* = 2, 58, *p* < .001), with no interaction with hemisphere (*F* = 0.28, *df* = 2, 58, *p* = .868). While the streamline count difference was originally driven by a greater proportion terminating in area V1, following normalization, the streamline count for area V1 was significantly lower when compared to the V2 (*t* = 4.51, *df* = 59, *p*_corr_ < .001) and V3 projections (*t* = 5.64, *df* = 59, *p*_corr_ < .001). No significant difference was observed between the V2 and V3 segments (*t* = 1.07, *df* = 59, *p*_corr_ = .864).

### False-positive rate estimation

In order to assess the validity of streamline count estimations, a control condition was introduced where the ratio of streamlines terminating in area V3A against those terminating in regions V1, V2 or V3 was used as an estimate of the false-positive rate of the tractography method. Under this condition, a significantly smaller proportion of streamlines terminated in the control site [Mean = 85 (4 %) ± 24 (1 %) SEM] compared to either area V2 [Mean = 427 (20 %) ± 76 (28 %) SEM; pairwise *t* test, *t* = 6.33, *df* = 59, *p* < .001] or V3 [Mean = 423 (20 %) ± 62 (23 %) SEM; pairwise *t* test, *t* = 8.86, *df* = 59, *p* < .001]. Furthermore, when the same data were normalized based on the surface area (mm^2^) of the corresponding target area, the hypothesized extrastriate projections to V2 (*t* = 7.18, *df* = 59, *p* < .001) and V3 (*t* = 9.80, *df* = 59, *p* < .001) retained a significantly greater proportion of streamlines when compared to the control area. This indicates that projections to areas V2 and V3 were not only larger than to the control site in real terms, as estimated by streamline count, but also in relative terms, while accounting for the cortical extent of the target region (see Fig. 5).

## Discussion

In this work, we identified extrastriate projections of the OR to areas V2 and V3 of occipital cortex in humans, in agreement with the macaque literature (Yukie and Iwai 1981; Benevento and Yoshida 1981; Fries 1981; Bullier and Kennedy 1983; Kennedy and Bullier 1985) and highlighting the anatomical homology of the retino-geniculate-striate pathway across primate species.

The OR projections to dorsal and ventral representation in occipital cortex follow anatomically distinct courses, with a predominance of upper visual field representation in the anterior bundle of the OR, in agreement with human dissection data (Ebeling and Reulen 1988; Wahler-Lück et al. 1991; Peuskens et al. 2004; Peltier et al. 2006) and a superior–inferior divide along the anterior-posterior segment. Similarly, the hierarchical projection segments of the OR follow anatomically segregated courses, with a medio-lateral nesting pattern. Both of these findings are consistent with the proposed hypothesis of distinct bundles connecting retinotopic LGN locations with a matching retinotopic representation in occipital cortex.

### Relative projection size

A larger V1 projection was observed bilaterally, when compared to connections with V2 and V3, as estimated by the relative number of streamlines terminating in each visual area. This finding is consistent with the idea of the OR as a primarily striate projection, but also with the overall larger cortical area representing V1, when compared to its neighboring maps V2 and V3 (Yan et al. 2009). We confirmed that cortical area size played an important role in this regard by examining the effects of normalization by target surface area on the observed streamline counts. The direction of the effect was in fact reversed, with areas V2 and V3 displaying proportionally larger number of streamlines terminating at those sites, when compared to the V1 projection. As evidenced here, projections to extrastriate areas V2 and V3 in fact account for a greater proportion of all OR streamlines per mm^2^ of cortical target zones.

Additionally, in order to provide an estimate of false-positive rate of the method implemented, we included a control site, V3A, to analyze the likelihood of generating streamlines to a cortical site where no direct thalamic projections were expected. In comparison to the control condition, projections to areas V2 and V3 were shown to be significantly larger, both in terms of number of streamlines reaching the target, and also when accounting for the surface area of the target zone. These results increase confidence on the V2 and V3 projections having a true anatomical basis and are unlikely to be false-positive connections arising as an artifact of the probabilistic nature of the tractography algorithm as indicated by the control region V3A that was examined.

### Microstructural findings

A hemispheric lateralization effect of OR microstructure was observed in all subjects tested, with lower FA and higher MD in the right, compared to the left OR (illustrated in Fig. 4). This result is consistent with the histological literature, where both the cortical (Bürgel et al. 1999) and the white matter area (Weinberger et al. 1982) of the left occipital region are found to be larger in the left than the right hemisphere. This hemispheric asymmetry would cause a detectable difference in microstructural measures, as larger, and more densely packed axonal bundles in the left hemisphere lead to increased FA and reduced MD, as reproduced in this study.

Perhaps more intriguing is the pattern of FA measured in hierarchy-based segmentations, where the left hemisphere displayed increased FA from V1 to V2 and V3, while the right hemisphere displayed and inverse pattern. An overall large tract volume in the left hemisphere, as previously noted, does not fully account for this observation and instead, it points toward a larger affordance of volume in the left OR sustaining a microstructurally robust V1 projection, while this affordance may be diminished in the smaller right OR. In real terms, the differential pattern of FA values across hierarchy-based segments of the OR is a small effect, as the variability between tract segments does not exceed FA < 0.05 in any single hemisphere. Nevertheless, such a pattern hints toward potential structural differences in white matter organization beyond binary hemispheric effects, and these findings suggest such variability may apply to specific segments of white matter pathways in isolation.

## Role of extrastriate projections

The existence of direct thalamo-cortical projections to extrastriate regions is perhaps not surprising; the visual system can be conceptualized as a hierarchical structure with a high degree of modularity, with distinct feature processing occurring in parallel and integrated to form a representation of visual information. As such, this system requires the provision of input; principally, but not exclusively, via the retino-geniculate pathway to multiple early regions for parallel processing and feed-forward to higher areas in occipital cortex and beyond. In addition, a large amount of connections along the OR are feedback connections, providing a bidirectional bridge between sub-cortical and cortical processing of visual information (Van Essen and Maunsell 1983; Felleman and Van Essen 1991). While it is not possible to distinguish feed-forward from feed-back projections with tractography techniques, direct extrastriate projections in human OR are likely to support both types of connection in V1 and at least feed-forward connectivity to extrastriate projections, in agreement with the structural arrangement seen in the macaque (Bullier and Kennedy 1983; Kennedy and Bullier 1985). In its entirety, the OR forms a substantial component in wider visual system, affording both parallel, bidirectional connections to multiple levels of cortical and sub-cortical areas involved in the processing of visual input.

## Conclusion

While typically described as a striate projection, the OR is hypothesized to connect the thalamus with extrastriate visual areas and in this study, we have identified and described direct OR projections to areas V2 and V3, in addition to striate cortex, V1 in humans in vivo. The arrangement of tract segments reveals anatomical segregation of upper and lower visual field representations into dorsal and ventral segments, as well as distinct white matter segments connecting to areas V1, V2 and V3 following a medio-lateral nesting pattern identifiable in single participants. Finally, microstructural differences between segments of the OR point to finer lateralization effects than previously considered, with significant differences in FA for V1 segments but not for extrastriate projections. Taken together, these findings point to a more nuanced functional arrangement of the human OR than previously considered, with the combination of functional and diffusion MRI allowing the tracing of specific eloquent segments of the white matter pathway into extrastriate regions.

## Figures and Tables

**Fig. 6:**
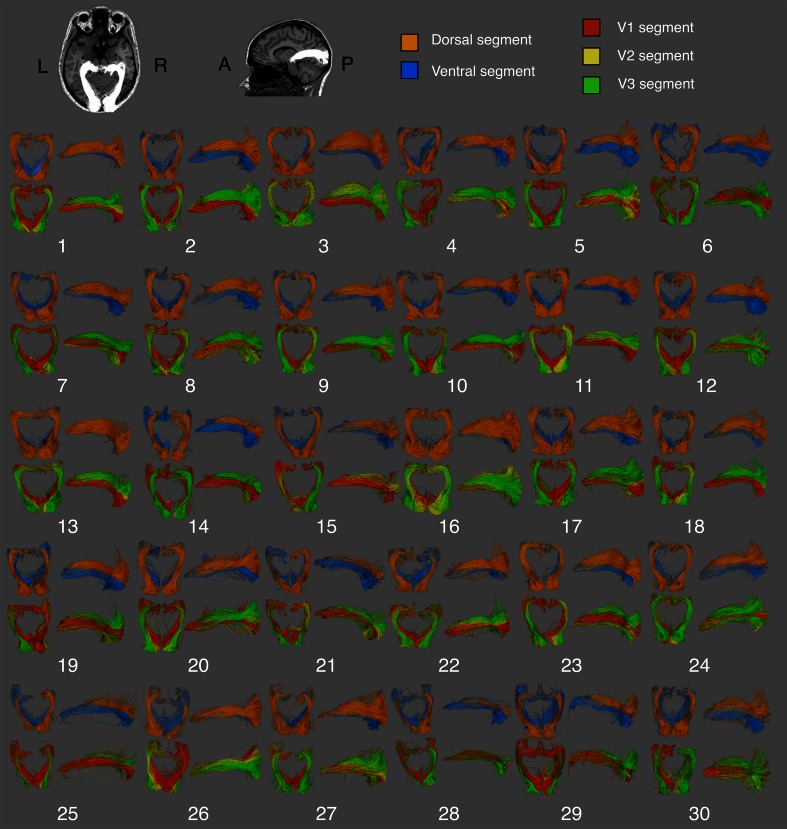
Two forms of segmentation for the optic radiation are presented in thirty participants; visual field-based segmentation, yielding dorsal and ventral projections and hierarchy-based segmentation, yielding *V1*, *V2* and *V3* projections. Two views are presented, an axial view from a superior vantage point, and a left-sided sagittal view from a lateral vantage point

**Fig. 7:**
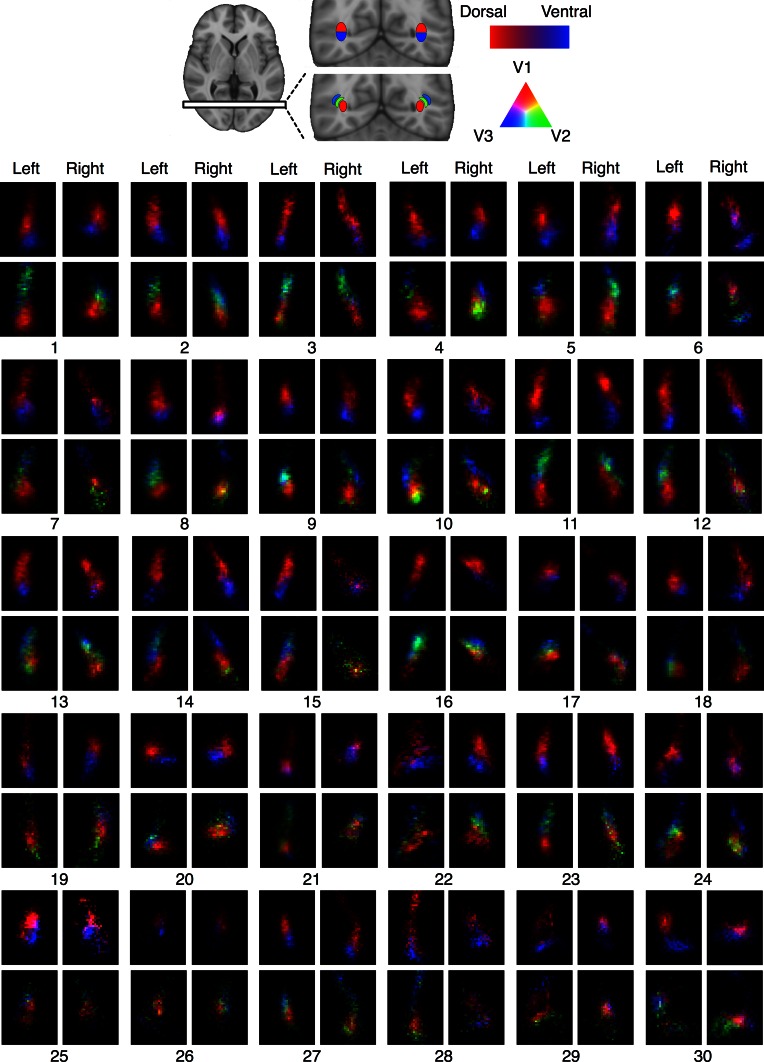
Visitation maps of optic radiation tractography in all thirty participants tested. All maps sampled at a single coronal slice immediately posterior to the lateral ventricle. Visual field-based segmentation shows the superior–inferior division of streamlines, in *red* and *blue*. Hierarchy-based segmentation discriminates streamlines into dorso–lateral nesting compartments, in *red*, *green* and *blue*

## Appendix 1

See Fig. 6.

## Appendix 2

See Fig. 7.
